# Useful Evidence by Post-Mortem CT and Stereomicroscopy in Bone Injury: A Case Series from Forensic Practice

**DOI:** 10.3390/healthcare11040540

**Published:** 2023-02-11

**Authors:** Giuseppe Davide Albano, Giovanni Scalzo, Ginevra Malta, Maria Puntarello, Alberto Alongi, Donatella Piscionieri, Giuseppe Francesco Lo Re, Sergio Salerno, Stefania Zerbo, Antonina Argo

**Affiliations:** 1 Section of Legal Medicine, Department of Health Promotion, Mother and Child Care, Internal Medicine and Medical Specialties, University of Palermo, I-90129 Palermo, Italy; 2 Radiology Unit, Department of Biomedicine, Neuroscience and Advanced Diagnostics, University of Palermo, Via del Vespro 129, I-90127 Palermo, Italy

**Keywords:** post-mortem CT, virtopsy, stereomicroscopy, bone injury, bones, burning, forensic, autopsy

## Abstract

The investigation of bone injuries is an essential topic in forensics. We sometimes have to deal with charred or dismembered human remains that have lost soft tissue, making it difficult to diagnose the mechanisms of injury that led to death. Our contribution aims to share with the scientific community how we approached two cases of very different bone injuries and the techniques used to differentiate relevant pathology findings of bone fragments. Two cases are analyzed from the case history of the forensic medicine institute of Palermo. PMCT made it possible to differentiate heat bone lesions from traumatic lesions. The stereomicroscope was superior to PMCT in being able to better characterize and evaluate shear injuries, allowing a more accurate measurement of acute lesions. Injury to bone remains can be appropriately investigated with rapid techniques such as PMCT and stereomicroscopy. The presented methodology shows the importance of a multidisciplinary approach when dealing with bone injuries in forensics that could find further applications in other circumstances to answer forensic questions.

## 1. Introduction

Bones are the body component most resistant to external damage. The bone tissue has a substantial dynamic capacity and can renew itself and change its form concerning the forces and pressures acting on it [1]. Understanding bone injury and its response to different damage have always been of great interest in clinical and forensic settings. The damages encountered in the cases illustrated are bladed injuries, traumatic injuries, and heat injuries. The damaging action of a blade on the bone results in V-shaped lesions with sharp apexes [2]. In the case of blunt injuries, it is helpful to identify the presence or absence of a linear transverse “V”-shaped shear section, trauma-associated fractures, loss or accumulation of tissue adjacent to the injury, or the presence of characteristic bone striations [3]. Exposure to a heat source causes collagen dehydration, resulting in a loss of bone elasticity that alters structural integrity and results in deformation and retraction of the bone surface [4,5]. Bone dehydration occurs at temperatures between 100 and 600 °C. It is always in the range of these temperatures that bone begins to deform and fracture [6]. During combustion, the bone changes its coloring. Several studies have shown that color variation can be associated with a specific exposure temperature [7,8,9]. In this regard, bone macroscopic analysis can provide information about circumstantial data and the manner of death.

Post-mortem CT (PMTC) has two significant advantages over conventional X-rays: three-dimensional image reconstruction and the ability to quantify X-ray attenuation. It is a non-invasive technique that allows images to be acquired in less than 15 min and is an essential guide to the subsequent autopsy investigation [10]. PMCT has proved very useful in studying bone lesions in charred corpses, allowing differentiation between heat-induced and traumatic fractures [11,12].

The presented cases show how the application ofa multidisciplinary approach involving the pathologist, the anthropologist, and the radiologist, by using PMCT and stereomicroscopic evaluation in two cases of present bone injury in a forensic context.

## 2. Case Series

### 2.1. Case 1

A dismembered body of a 47-year-old woman was found in the countryside. The dismembered remains had an overall length of 101 cm. The finding as a whole consisted of the lower half of the human body, with remnants of the vertebral column, pelvis, and lower limbs. External inspection showed signs of fraying and substance loss at the first lumbar vertebra level, while the subsequent vertebrae were perfectly fused. The bony surfaces and intervertebral discs were diffusely irregular, characterized by diffuse areas of loss of substance and dimpling. At the anterolateral right side of the soma of the third lumbar vertebra, there was a linear, central horizontal axis, sharp-edged wound, approximately 2.5 cm in length, which deepened into the bone tissue by a few millimeters. The surface of the left iliac crest was irregular and jagged due to the action of the macrofauna. Two acute-angled bone incisions were also present.

### 2.2. Case 2

A 16-year-old man was found dead in his car following a car accident. His body was found charred inside the car. On external inspection, the corpse showed a fracture of the cranial vault from a burst with exposure of the brain mass; injury to the vertex; disintegration of the parietal bones; silvery discoloration of the outer edge of the right orbit, and the residual part of the nasal pyramid; amputation at the level of the lower third of the right arm, the upper third of the left forearm, as well as at the middle third of the right thigh, the upper third of the left leg; fracture of the right tibia; initial greyish-black discoloration of the exposed stump of the middle third of the right thigh; a residual fraction of femur and tarsal bones with tendon exposure.

The autopsy showed multiple right costal fractures at the contralateral end of the sternal dumbbell. The heat burns also resulted in burst fractures involving the bones of the neuro- and splanchnocranium, as well as the upper and lower limbs; in addition to these fractures, there were traumatic injuries involving the thoracic and pelvic-sacral region.

### 2.3. PMCT Finding

**Figure 1:**
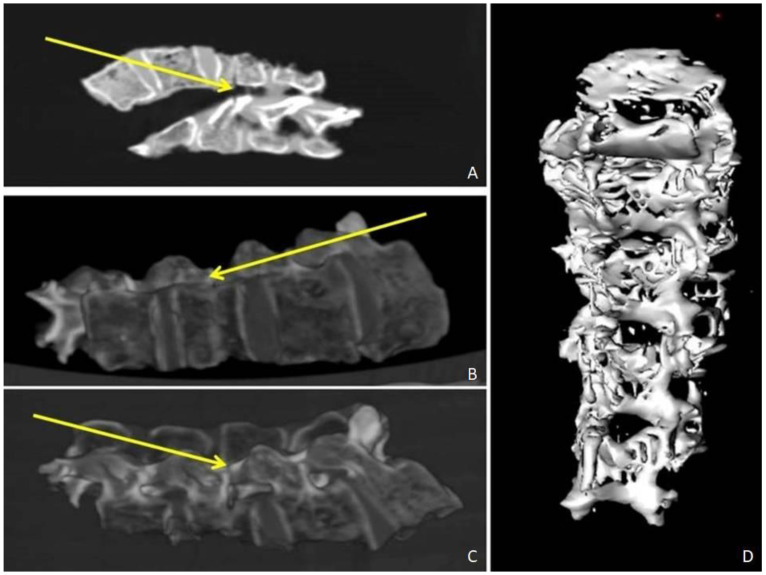
Case 1. PMCT Coronal plane reconstruction. (**A**–**C**) Axial PMCT planes show disruption of the cortical bone at the vertebral processes (yellow arrows). (**D**) Three-dimensional volume rendering reconstruction.

**Figure 2:**
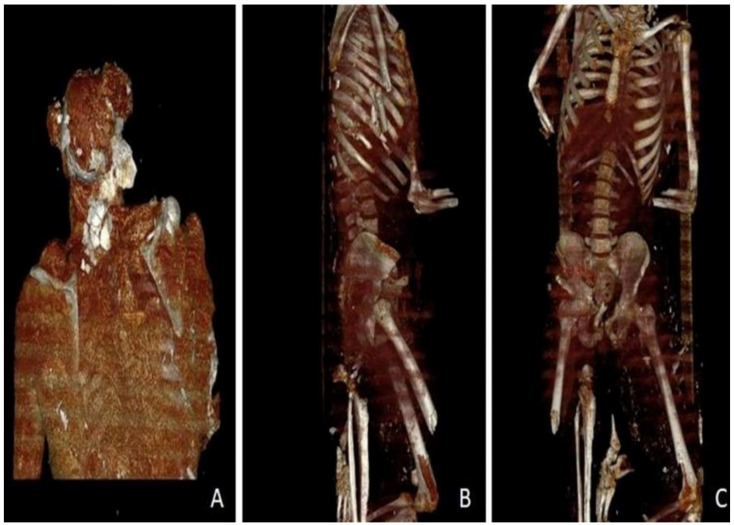
Case 2. Three-dimensional VR coronal deformatted images made by the dedicated workstation. (**A**) Burst fracture of the cranial theca. (**B**,**C**) Flutemouthpiece of the long bones and thermal delamination of the cortical of the left femur.

**Figure 3:**
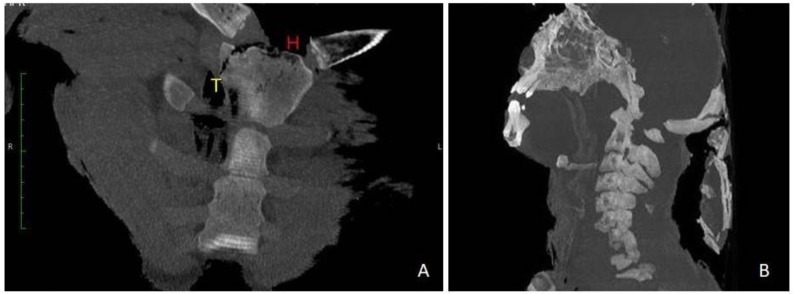
Case 2. (**A**) Paracoronal reformatted MIP-SLAB (20 mm). T: traumatic fracture; H: heat fracture. (**B**) Sagittal reformatted MIP-SLAB (50 mm). Explosion of the cranial vault.

Before the autopsy, PM-CT was carried out on 128-section multidetector CT scanners (Somatom Definition AS, Siemens Healthcare, Erlangen, Germany). The CT protocol consisted of a single total-body acquisition (nominal thickness 0.625 mm, reconstruction thickness 0.625 mm, and 1.25) In case 1, the three-dimensional CT reconstruction showedthe presence of shearing lesions at the level of the third lumbar vertebrae and the disruption of the cortical bone at the vertebral processes (Figure 1). In case 2, total-body CT scan showed multiple fracture rims with clean margins of the neurocranium, upper and lower limb fractures, multi-fragmented fracture of the right zygomatic bone and the nose, and fracture of the sternum; right costal fractures of II, IV, V, VII, and VIII;fracture of the right iliac spine, the right sacral half, the ischial branches, and the pubic bones bilaterally (Figure 2 and Figure 3). PMCT permitted to distinguish traumatic from heat fractures.

### 2.4. Stereomicroscope

**Figure 4:**
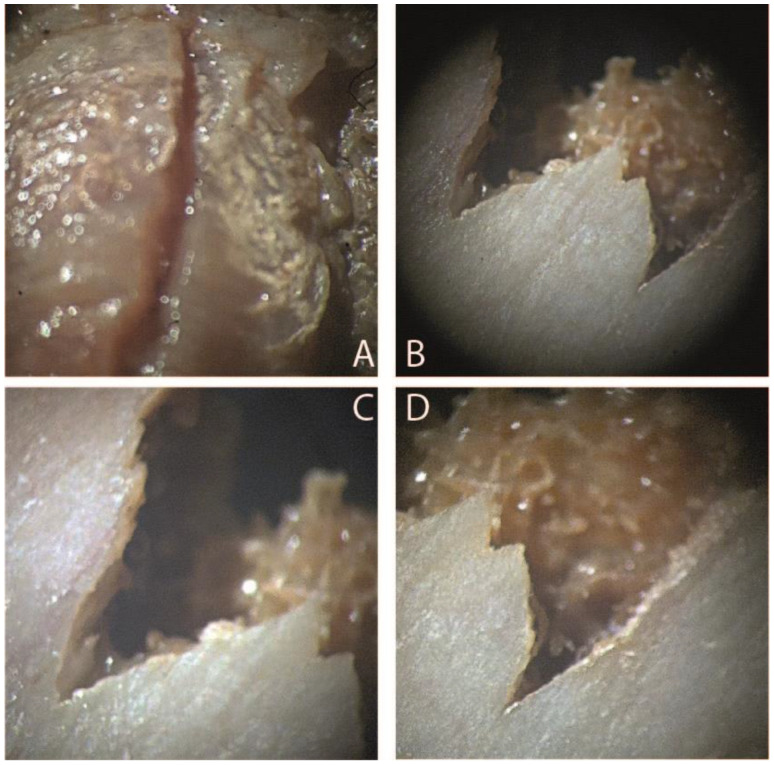
View of bone lesions with stereomicroscopy (Carl Zeiss A772415, CarlZeiss Co., Oberkochen, Germany) of case 1 (scale bar 5:1). (**A**) Lesion at the level of the III lumbar vertebra (zoom ×3.2). It appears linear, with clean margins, perfectly referable to the action of a blade; (**B**) lesions at the level of the iliac wing (zoom ×3.2). The classic ‘V’ shape and sharp margins can be seen. (**C**,**D**) Detail of the “V” shape (zoom ×3.6).

**Figure 5:**
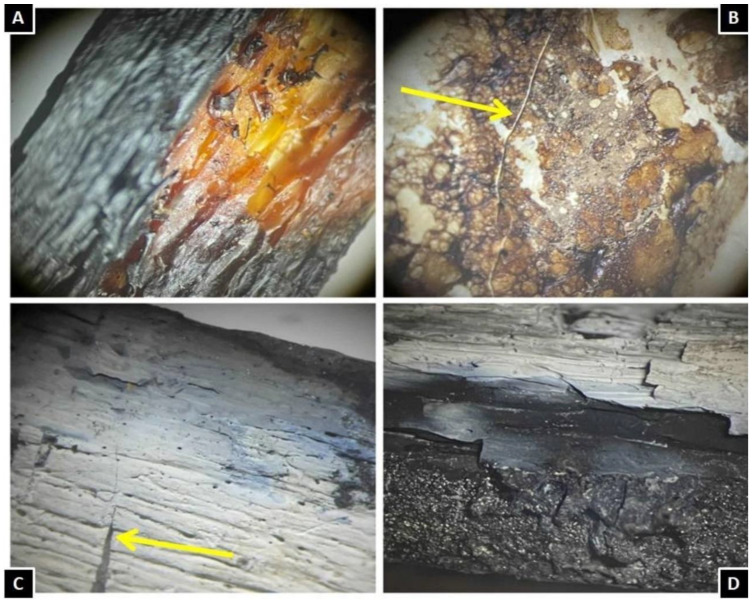
Case 2. Stereo microscopy view of several bone fragments. (**A**) Femur diaphysis: typical staining from exposure to a temperature of around 300/400 °C (brown-greyish-reddish) (**B**). Cranial fragment: the yellow arrow indicates the typical heat bone fracture, characterized by being thin and with regular margins. (**C**) Cortical of long bone: the bluish-white-grey coloring, the deep heat fissuring (indicated by the yellow arrow) and the thin cracks (outer third of the bone cortical) due to the effect of loss of organic matrix of the bone under the effect of heat can be appreciated in detail. (**D**) Phenomenon of delamination of the cortical bone.

In the laboratory, the bones were cleaned first by brushing and then washed with water with sieving of the water used for washing to avoid losing any valuable finds. Subsequently, bone fragments of the most significant interest were observed through a stereomicroscope(Carl Zeiss A772415, Carl Zeiss Co., Oberkochen, Germany) to determine the presence or absence of cut mark characteristics. In case 1,the lesion at the level of the intervertebral disc of the III vertebra appears linear, with an accumulation of tissue adjacent to the lesion and perfectly compatible with a stab lesion of a depth of 4 mm. The lesions at the level of the iliac crest present a “V” shape, a classic form of stabbing lesions(Figure 4). In case 2, the color variations of the charred remains were assessed using the Munsell, HEX, and RGB color scales to extrapolate the different exposure temperatures. The femur diaphysis, the cranium, and the long bones showed the typical changes of burned bones at stereomicroscopy analysis: color changes, fissuring, cracking, fractures, delamination of the cortical bone (Figure 5).

## 3. Discussion

The conventional autopsy has limitations that can be overcome through ancillary investigations [13].

When encountering stab wounds in forensic practice, a significant aid is provided by stereomicroscopy.A stereomicroscopeis is an inexpensive instrument readily available in forensic laboratories. It can be beneficial to differentiate a cut mark from a blunt trauma: regular edges due to the sharp mechanism and irregular edges due to the blunt mechanism [1,3]. A blunt weapon generates unstable, non-specific fractures. A blade generates typical striations on bone and cartilage. In addition, fractures are usually generated at the points of least resistance and are easily distinguishable from sharp injuries [14].

The action of a blade on a bone can generate hinge fractures. They are formed as a consequence of the retracting movement of the knife. This movement leads to a change in the orientation of the handle, which can prove a hinge fracture defect on the side of the cut mark opposite to the handedness of the individual [15]. Bernani et al. compared the use of the stereomicroscope and the electron microscope (SEM) in the study of false saw start. When comparing five parameters: the shape of the profiles, the shape of the walls, the pattern of the striae of the kerf floor, the shape of the bone islands, and the presence of bone debris, the use of the SM was equivalent in some of them [16]. Using a stereomicroscope to examine cut marks on bone may be sufficient in most cases. Regarding the burned body, the stereoscope investigation made it possible to isolate the different color shades of the cortical and thus extrapolate the average exposure temperatures(Table 1). Moreover, in case 2, stereomicroscopy analysis of burned bones showed the typical alterations of bones subjected to heat. Microscopic study is recommended in those cases where the usual macroscopic burning indicators are ambiguous or difficult to determine. In addition to temperature, other variables can influence the coloring of the bone. These are due to the presence or absence of organic material around the bone during combustion, discharge duration, or oxygen availability. Walker et al. showed that the availability of oxygen and the presence of organic material in the soil and oxygen availability are the factors that most influence bone color changes. Although important, the duration of heat action on the bone plays a minor role in color change. A recent experimental study showed that the color differences were minimal in bones charred after one hour and three hours. Moreover, this study stated that the duration of the burning process had less influence on bone color than other variables (temperature and environmental factors) [7]. In this regard, consistent data about the effects of the duration of burning on bone features are limited in the literature. As we mentioned before, one of the factors that influences the transformation of burnt bone is the presence of oxygen, therefore concerning open, airy environments rich in greater availability of vegetable oxygen. In the presence of oxygen, the temperatures reached are much higher, and therefore, the burnt bones’ color change is faster. The range of colors observed in charred bones is different in the absence of oxygen. A variation of colors is observed, which is slowed down. In carbonized bone (lacking oxygen), the color varies from white to white-yellow and yellow to red-brown at 300 °C. After this phase, at 600 °C, the color of the bone is black, while at 900°, and the bone remains black with reddish hues. Higher temperatures are appreciated more quickly in the bone burned in oxygen-rich environments and in the open air. At 350 °C, the bone becomes dark red-brown. From 400 to 600°, it becomes gray, while at temperatures higher than 900–1000 °C, the color of the bone becomes bright white [9,17,18,19]. The stereomicroscopy made it possible to evaluate the different bone colors due to exposure to a heat source [7,8,9]. In particular, the dominant colors ranged from red to brown. In some places, variations of grey and black were found.

On the other hand, a femoral fragment showed bluish-grey color, suggestingan exposure at about 500°/700°. A fracture could explain the colorimetric difference from other bones due to the trauma of the accident before heat exposure. The heat could have acted to a greater extent on this fragment not being covered and protected by soft tissues. In other fragments, the temperature was estimated at about 300°.

PMCT is essential in the case of knife injuries when the corpse is not dismembered [20]. Another tool in a forensic setting is the use of magnetic resonance imaging (MRI). It is used as a complement to PMCT investigation as it is excellent at recognizing soft tissue contrast. MRI allows the study of fluid accumulation at the soft-tissue level. However, in forensic analysis, decomposing bodies show extensive fluid collections in dependent subcutaneous fat areas, which may mask post-traumatic findings and produce false-positive MRI results [21]. MRI is recommended to diagnose sudden cardiac death or soft tissue trauma such as stab wounds, blunt force, or age determination [22]. In our cases, PMCT, stereomicroscope, and autopsy were sufficient in discriminating the different injuries. Indeed, PMCTmakes it possible to assess the blade’s path inside the body, in some cases providing more information than autopsy alone. Our case, however, involved dismembered remains. The 3D reconstruction showed the presence of a stab wound to the third vertebra and the destruction of the bony cortical. Through autopsy, these injuries were easily visible.

PMCT has proven useful, especially in traumatic injuries following road accidents [10,23,24]. Forensic medicine is interested in the study of accidents to describe the events that caused the damage, and the reconstruction of accidents and cause and manner of death is essential [25]. When the vehicle catches fire after an accident, it can be difficult to differentiate the pathophysiology of fractures. In this regard, three-dimensional reconstruction of bone fractures can improve diagnostic accuracy [26,27].

Another critical point is the age of the victims. The bones of young people have a different response to heat and blunt trauma than older bones. A recent experimental study showed that younger bone may fragment less than older bone because it can withstand more significant deformation before reaching the fracture point threshold [28]. Even before the autopsy, PMCT can make it possible to differentiate traumatic injuries from heat injuries based on their morphology [13,29,30]. The traumatic bone fractures can be distinguished from heat fractures as they are multifractured and less organized. Heat fractures, on the other hand, show clear margins or clear amputations. Heat fractures of the cranial theca in case 2 were evident: CT scan reported heat fractures as linear and with clear margins.According to literature trans-diploid fractures correspond to the separation of the inner and outer plates of the skull under the action of heat. Moreover, trans-diploid fractures are characterized by the separation of the inner and outer plates of the skull under the action of heat, and by a longitudinal fracture line that crosses the diploe parallel to the cortical bone. Differently, traumatic fractures typically affect both the inner and outer planks and are perpendicular to the bone’s surface.On extensively charred bodies, a complete split between the bone planks is possible, or even the disappearance of the outer bone plank under heat as areas of superficial delamination. Fractures or amputations of long bones have the typical blunt ends described as ‘flute-mouthpiece’ appearance [31,32].

Alongside the case 2 fractures were bony heat amputations of the upper and lower limbs. Heat fractures are thin and linear. If the extremities of the limbs are affected, heat amputations can be observed with regular margins. Such amputations differ from traumatic amputations because they are not covered by soft tissue or skeletal muscle [13]. Furthermore, in some cases, fragmentation can be observed. In our case, PMCT highlighted traumatic fractures involving the eyelid rhyme, sternum, ribs, and pelvic bones. Heat fractures were highlighted at the cranium and the upper and lower limbs.

## 4. Conclusions

Three-dimensional reconstruction made it possible to highlight and better characterize bone fracture and guide the subsequent autopsy investigation. In case of stab wounds, the use of stereomicroscopy can be essential to highlight several signs such as contusion or the precise size of bone lesions, guaranteeing a more excellent characterization of the presented bone injuries. The combined use of PMCT and stereomicroscopy is also helpful in the case of charred subjects. Stereomicroscopy, due to greater precision regarding the exposure temperature of the bone, allows a study of the variations in color according to heat and environmental factors.The burned bone undergoes organic changes also involving density, which could be investigated in the future by PMCT investigation to provide further information for forensic purposes. In the presented cases, the combined use of PMCT and stereomicroscopy was essential to evaluate the bone injuries. The presented methodology demonstrates the importance of a multidisciplinary approach when dealing with bone injuries in forensics that could find further applications in other circumstances to answer forensic questions.

## Figures and Tables

**Table 1:** Literature on the colorimetric variations tosuggest the exposure temperature of the bone fragments.

T in °C	Walker, Miller, and Richman, 2008 [7]	Absolonovà, Dobisikovà, Beran, Zocovà, and Veleminsky, 2012 [8]	Ellingham, Thompson, Islam, and Taylor, 2014 [9]
100	Brownish	Yellowish	Yellow-white
200	Brown	Grey-brown or dark brown	Yellow-white
300	Black	Dark brown to black	Brown-black-reddish-dark grey
400	Brown-greyish	Dark brown to black	Brown-black-reddish-dark grey
500	Dark grey	Light grey/light blue grey	Grey-black-blue/grey-reddish yellow spots
600	Grey	Tending to white	Grey-black-blue/grey-reddish yellow spots
700	Light grey	Bright white, greyish-white, bluish-grey or dark grey	Milk white-grey/blue spots
800	Ever lighter grey	White	White

## Data Availability

All data are included in the main text.
